# Single-Cell RNA Sequencing Identifies Intra-Graft Population Heterogeneity in Acute Heart Allograft Rejection in Mouse

**DOI:** 10.3389/fimmu.2022.832573

**Published:** 2022-02-10

**Authors:** Yunhua Tang, Jiali Wang, Yixi Zhang, Jun Li, Maogen Chen, Yifang Gao, Meiqin Dai, Shengjie Lin, Xiaoshun He, Chenglin Wu, Xiaomin Shi

**Affiliations:** ^1^ Organ Transplant Center, The First Affiliated Hospital, Sun Yat-sen University, Guangzhou, China; ^2^ Guangdong Provincial Key Laboratory of Organ Donation and Transplant Immunology, Guangzhou, China; ^3^ Guangdong Provincial International Cooperation Base of Science and Technology (Organ Transplantation), Guangzhou, China; ^4^ Department of Nephrology, The First Affiliated Hospital, Sun Yat-sen University, Guangzhou, China

**Keywords:** transplantation, acute rejection, macrophages, endothelial cells, single-cell RNA sequencing

## Abstract

Transplant rejection remains a major barrier to graft survival and involves a diversity of cell types. However, the heterogeneity of each cell type in the allograft remains poorly defined. In the present study, we used single-cell RNA sequencing technology to analyze graft-infiltrating cells to describe cell types and states associated with acute rejection in a mouse heart transplant model. Unsupervised clustering analysis revealed 21 distinct cell populations. Macrophages formed five cell clusters: two resident macrophage groups, two infiltrating macrophage groups and one dendritic cell-like monocyte group. Infiltrating macrophages were predominantly from allogeneic grafts. Nevertheless, only one infiltrating macrophage cluster was in an active state with the upregulation of *CD40*, *Fam26f* and *Pira2*, while the other was metabolically silent. Re-clustering of endothelial cells identified five subclusters. Interestingly, one of the endothelial cell populations was almost exclusively from allogeneic grafts. Further analysis of this population showed activation of antigen processing and presentation pathway and upregulation of MHC class II molecules. In addition, Ubiquitin D was specifically expressed in such endothelial cell population. The upregulation of Ubiquitin D in rejection was validated by staining of mouse heart grafts and human kidney biopsy specimens. Our findings present a comprehensive analysis of intra-graft cell heterogeneity, describe specific macrophage and endothelial cell populations which mediate rejection, and provide a potential predictive biomarker for rejection in the clinic.

## Introduction

Organ transplantation is a preferred choice of treatment for patients with end stage diseases. Despite advances in the field, rejection remains a major barrier to both short-term and long-term graft survival. Currently, immunosuppressive drugs targeting T cells are widely used in the clinic, which effectively suppresses acute rejection and significantly improves short-term graft survival. Unfortunately, acute rejection still occurs even when T cells are depleted in some cases, indicating that non-T cell factors are involved in graft rejection (1, 2).

Rejection is a very complex process involving the participation and coordination of all types of immune cells and non-immune cells. Conventional methods, such as microarray and bulk RNA sequencing, have been extensively applied to explore the regulation network associated with rejection (3, 4). Our recent work reported a profile of lncRNAs in acute rejection which may provide potential diagnostic markers in the clinic (5). However, in the rejection response, information acquired by traditional assays is incomplete, especially those related to features of each specific cell type. Recently, techniques for single-cell RNA sequencing (scRNA-seq) have been developed and optimized which allow rapid and simultaneous detection of thousands of genes at the single-cell level. scRNA-seq has revolutionized our way to study the complexity of different cell types in an environment (6, 7). With scRNA-seq, Wu et al. successfully identified 16 cell types in a single human kidney allograft biopsy and found diverse immune cell infiltrates as well as novel endothelial cell states (8). However, due to limited sample size of kidney biopsy and the high heterogeneity of human samples, the results might be incomplete and skewed. Therefore, we chose to take advantage of scRNA-seq to study acute rejection in the well-established mouse heart transplant model.

In this study, we identified 21 cell populations. As expected, we observed known pathways associated with rejection, such as activation and expansion of T cells and NK cells. We also characterized heterogeneity of graft infiltrating macrophages. Importantly, we identified a novel endothelial cell subset which can function as antigen presenting cells that potentially mediate transplant rejection. The expression of Ubiquitin D was significantly higher in such endothelial cells, which was confirmed in the mouse allogeneic heart grafts and in the kidney biopsy specimens with rejection.

## Materials and Methods

### Animals

Male C57BL/6J and BALB/c mice aged eight to ten weeks were obtained from the Animal Experimental Center of Sun Yat-sen University. All animal care and experiments were approved by the Animal Care Ethics Committee of the First Affiliated Hospital of Sun Yat-sen University.

### Heterotopic Cardiac Transplantation

Mouse heterotopic heart transplantation was performed as described in our previous study (9). Briefly, donor heart grafts were harvested from C57BL/6J mice or BALB/c mice and then transplanted into the abdominal cavity of recipient C57BL/6J mice *via* anastomosing the ascending aorta and pulmonary artery of the graft end-to-side to the recipient’s aorta and vena cava respectively. Daily transabdominal palpation was used for assessing graft survival, and graft rejection was defined as complete cessation of palpable heartbeats, confirmed by laparotomy. Heart grafts were harvested on Day 5 post-transplant for single-cell transcriptional profiling.

### Clinical Samples

Formalin-fixed, paraffin embedded kidney biopsy specimens were collected from 10 patients who accepted kidney transplantation at the First Affiliated Hospital of Sun Yat-sen University between July 2015 and August 2018. All 10 donors were enrolled in a voluntary organ donation program in China. The study procedure was approved by the Research Ethics Committee of the First Affiliated Hospital of Sun Yat-sen University. Informed consent was obtained from every patient included in the study. The clinicopathologic variables of the patients are as shown in **Supplementary Table 1**.

### Single-Cell Dissociation

Single-cell RNA-seq experiments were performed in the laboratory of NovelBio Bio-Pharm Technology Co., Ltd. The mouse heart allografts were surgically removed and kept in MACS Tissue Storage Solution (Miltenyi Biotec) until processing. The tissue samples were processed as described below. In brief, samples were first washed with phosphate-buffered saline (PBS), minced into small pieces on ice and enzymatically digested with MACS containing150 U/mL collagenase II (Worthington), 275 U/mg collagenase IV (Worthington), 1.2U/mL dispase II (Roche) and 50 U/mL DNase I (Worthington) for 45 mins at 37°C (agitation twice). After digestion, samples were filtered through a 70µm cell strainer, and centrifuged at 300g for 5 min. After the supernatant was removed, the pelleted cells were suspended in red blood cell lysis buffer (Miltenyi Biotec) to lyse red blood cells. After washing with PBS containing 0.04% BSA, the cell pellets were re-suspended in PBS containing 0.04% BSA and re-filtered through a 35μm cell strainer. Dissociated single cells were then stained with AO/PI for viability assessment using Countstar Fluorescence Cell Analyzer.

### Single-Cell Sequencing

The scRNA-seq libraries were generated using the 10X Genomics Chromium Controller Instrument and Chromium Single Cell 3’ V3 Reagent Kits (10X Genomics, Pleasanton, CA) according to the standard protocol provided in the manual. In brief, cells were adjusted to 1000 cells/uL and loaded into each channel to generate single-cell Gel Bead-In-Emulsions (GEMs). After the RT step, GEMs were broken and barcoded cDNA was purified and amplified. The amplified barcoded cDNA was fragmented, poly A-tailed, ligated with adaptors and index PCR amplified. The final libraries were quantified using the Qubit High Sensitivity DNA assay (Thermo Fisher Scientific) and the size distribution of the libraries were determined using a High Sensitivity DNA chip on a Bioanalyzer 2200 (Agilent). All libraries were sequenced by Illumina Sequencer (Illumina, San Diego, CA) on a 150 bp paired-end run.

### Single-Cell Sequencing Data Processing

scRNA-seq data analysis was performed by NovelBio Bio-Pharm Technology Co., Ltd. with NovelBrain Cloud Analysis Platform. Cellranger software (version 3.0.0) was used to generate fastp (10) files with default parameters and to align reads to the mouse genome (mm10 Ensemble: version 92). Cells containing over 200 expressed genes with mitochondria UMI rate below 20% were kept and mitochondria genes were removed. We applied the doubletCell function in the scran package to mark doublet cells and set the percentage of doublet cells at 5%.

Seurat package (version 2.3.4, https://satijalab.org/seurat/) was used for data normalization and analysis. Principal Component Analysis (PCA) was generated based on the scaled data with top 2000 highly variable genes. The first 10 principal components were selected as input for t-distributed stochastic neighbor embedding (tSNE) construction and UMAP construction. We used the function “FindAllMarkers” in Seurat to identify differentially expressed genes in each cell cluster with Wilcox rank sum test algorithm under the following criteria: 1. lnFC > 0.25; 2. P value<0.05; 3. min.pct>0.1. For further exploration, cells of the same cell type were selected for re-tSNE analysis, graph-based clustering and marker analysis.

### Gene Ontology Analysis

We downloaded the GO annotations from NCBI (http://www.ncbi.nlm.nih.gov/), UniProt (http://www.uniprot.org/) and Gene Ontology (http://www.geneontology.org/) (11). Fisher’s exact test was applied to identify significant GO categories and FDR was used to correct the P values.

### Pathway Analysis

We performed pathway analysis according to KEGG database. We used Fisher’s exact test to select significant pathways, and the threshold of significance was defined by P value and FDR (12).

### Quantitative Set Analysis for Gene Expression Analysis

QuSAGE analysis was performed as described (13).

### Gene Co-Regulation Analysis

We used the “find_gene_modules” function of Monocle 3 with default parameters to identity gene co-regulation network (14).

### Immunohistochemistry

After deparaffinization with dimethylbenzene, tissue sections were sequentially rehydrated with graded alcohols. The antigens were retrieved by boiling the sections in citrate–disodium hydrogen phosphate buffer (pH 6.0) with high pressure for 5 minutes. Endogenous peroxidase was inactivated by incubation with 0.3% hydrogen peroxide for 30 minutes. The slides were subsequently incubated with the primary antibody against FAT10 (ab134077, Abcam, USA) overnight at 4°C, incubated with the biotinylated secondary antibody and streptavidin peroxidase (Invitrogen, Grand Island, USA) for 30 minutes at 37°C, and developed with 3,3′-diaminobenzidine solution (GeneTech, Shanghai, China) before counter-staining with hematoxylin.

### Statistical Analysis

Statistical analysis was performed using SPSS v19.0 software (Chicago, IL, USA). Student t test or Wilcoxon rank-sum test was used. A value was considered statistically significant if P<0.05.

## Results

### scRNA-seq Identifies 21 Distinct Cell Types in Mouse Heart Allograft

**Figure 1:**
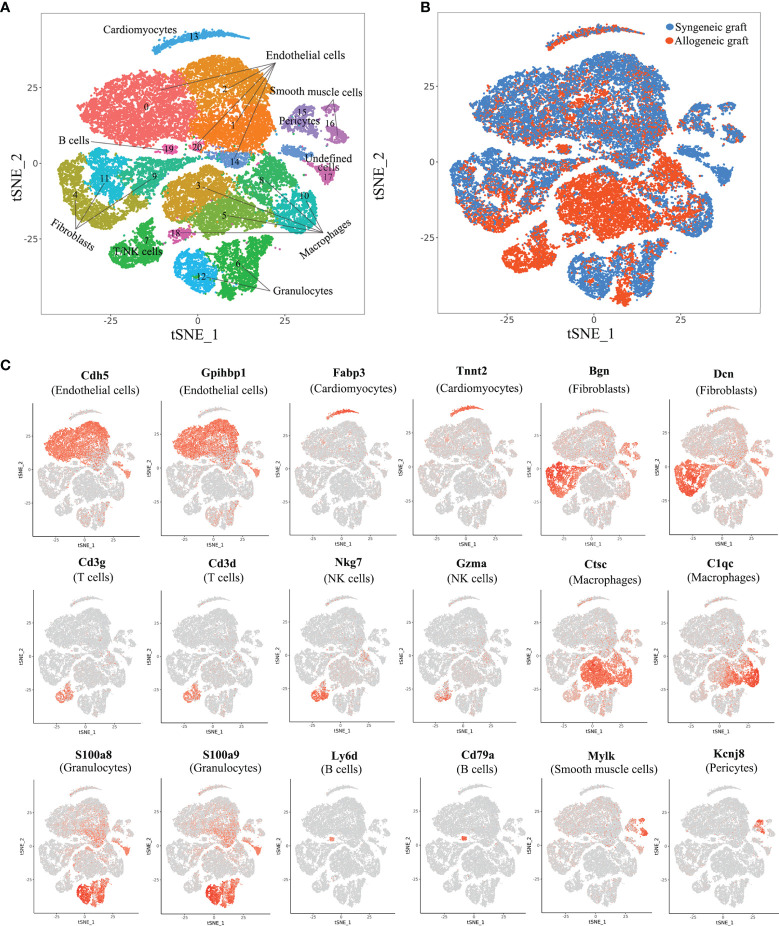
Comprehensive scRNA-seq analysis of cell types in syngeneic and allogeneic heart grafts. **(A)** Single-cell transcriptomes of cardiac cells in two syngeneic and two allogeneic heart grafts collected on Day 5 post-transplant were analyzed with an unsupervised dimensionality reduction algorithm (Seurat) to identify groups of cells with similar gene expression pattern. Each dot stands for a cell. **(B)** Distribution of cells originating from syngeneic graft or allogeneic graft as indicated in **(A)**. **(C)** Canonical cell markers were used to characterize cell clusters in the t-SNE plot, including endothelial cells, cardiomyocytes, fibroblasts, T cells, macrophages, granulocytes, B cells, smooth muscle cells and pericytes.

To comprehensively analyze cell populations in acute rejection at the single-cell level, we performed 4 cases of heterotopic mouse heart transplantation (2 syngeneic and 2 allogeneic) and collected grafts on Day 5. For each graft, single-cell RNA profiling was performed using the 10x Chromium platform. In an initial quality control, on average, we detected transcripts from 2,033 different genes with 51,882 sequencing reads per cell in the merged data set. In total, we acquired 18,698 cells from 2 allogeneic heart grafts and 19,904 cells from 2 syngeneic heart grafts (**Supplementary Figure 1**). With unsupervised clustering analysis using Seurat based on shared and unique patterns of gene expression, we identified 21 distinct cell clusters (**Figure 1A**). As expected, syngeneic grafts and allogeneic grafts displayed different patterns of cell populations (**Figure 1B**). We annotated cell clusters in accordance with signature gene expression and literature. In essence, we identified cardiomyocytes (1.80%, *Tnt2* ^+^, *Fabp3* ^+^), five types of endothelial cells (46.52%, *Cdh5* ^+^, *Gpihbp1* ^+^), three fibroblast populations (26.29%, *Dcn* ^+^, *Bgn* ^+^), T/NK cells (1.36%, *Cd3d* ^+^, *Cd3g* ^+^, Nkg7^+^, Gzma^+^), five types of macrophages (14.01%, *C1qc* ^+^, *Ctsc* ^+^), two granulocyte populations (3.27%, *S100a9* ^+^, *S100a8* ^+^), B cells (1.24%, *Ly6d* ^+^, *Cd79a* ^+^), smooth muscle cells (2.09%, *Mylk* ^+^), pericytes (3.37%, *Kcnj8* ^+^) and an undefined cell population (0.05%, unknown) (**Figures 1A, C**). The full set of raw data of this study has been deposited in NCBI’s Gene Expression Omnibus (GEO) and is available through the GEO accession number GSE151048.

### Activation of T Cells and NK Cells in Allograft Rejection

**Figure 2:**
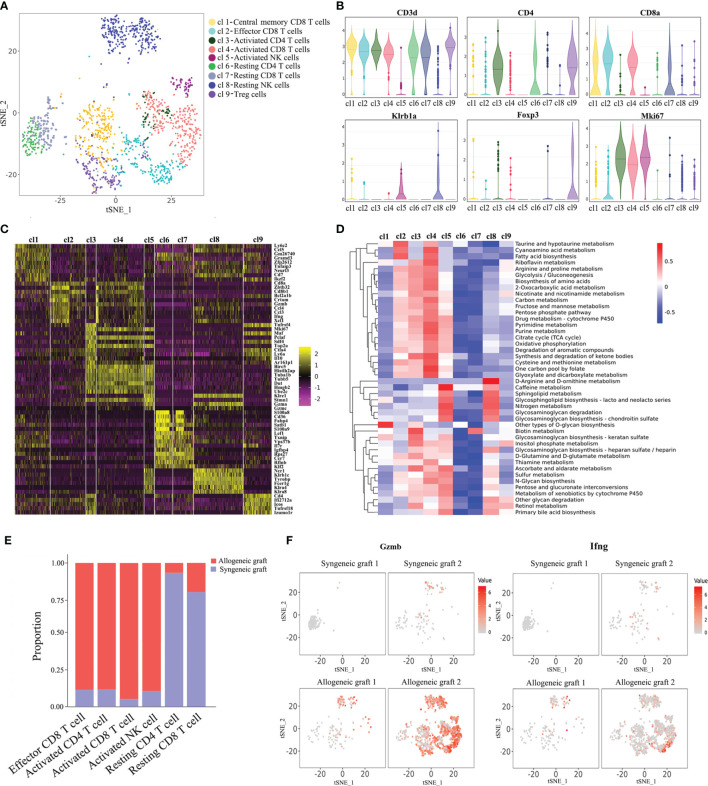
Characterization of T cells and NK cells. **(A)** Reclustering of T/NK cells with the t-SNE plot identifying seven T cell populations and two NK cell populations. **(B)** Violin plots displaying the expression of representative cell markers (CD3d for T cells, CD4 for CD4^+^ T cells, CD8a for CD8^+^ T cells, Klrb1a for NK cells, Foxp3 for Treg cells, Mki67 for proliferating cells). The y axis shows normalized read counts. **(C)** Heatmap of all T/NK cells clustered by recursive hierarchical clustering and Louvain–Jaccard clustering (Seurat) showing gene expression signatures in each cell population. **(D)** Quantitative Set Analysis for Gene Expression (QuSAGE) analysis examining active cellular metabolic pathways in each T/NK cell population. **(E)** Proportion of cells in each T/NK cell cluster from allogeneic or syngeneic graft. **(F)** Expression of Gzmb and Ifng in the allogeneic and syngeneic heart grafts in the t-SNE plots as shown in **(A)**.

It is known that T cells and NK cells play a significant role in acute rejection. We made further analysis of T/NK cells with single-cell transcriptomes. Re-clustering of T/NK cells revealed seven T cell populations and two clusters of NK cells (**Figure 2A**). Cluste1,2,4,7 were characterized as CD8+ T cells due to the expression of CD8a. We also observed that Cluster 1 expressed genes previously associated with central memory T cells (Lef1, Cd69, Tcf7), and Cluster 2 expressed genes associated with effector T cells (Zeb2, Tnf, Ifng) (**Supplementary Figure 2**) (15). Combining the expression of signature marker genes, cell-proliferating gene (*Mki67*) and differentially expressed genes, we annotated these cell clusters as Central memory CD8^+^ T cells, Effector CD8^+^ T cells, Activated CD4^+^ T cells, Activated CD8^+^ T cells, Activated NK cells, Resting CD4^+^ T cells, Resting CD8^+^ T cells, Resting NK cells and Treg cells (**Figures 2A–C**). Additionally, a large number of metabolic pathways were stimulated in Activated CD4^+^ T cells, CD8^+^ T cells and NK cells, such as glycolysis/gluconeogenesis pathway, oxidative phosphorylation pathway, pyrimidine metabolism pathway and purine metabolism pathway (**Figure 2D**), which are involved in graft rejection (16). Unsurprisingly, Activated T cells and NK cells predominantly comprised cells from allogeneic grafts, whereas Resting T cells predominantly comprised cells from syngeneic grafts (**Figure 2E**). Furthermore, we found enhanced expression of granzyme B (*Gzmb*) and interferon-γ (*Ifng*) in allogeneic heart grafts (**Figure 2F**), which is consistent with previous reports (17, 18).

### Macrophage Heterogeneity in Allograft Rejection

Currently, most immunosuppressive drugs target T cells. However, more and more studies demonstrate that innate immune cells play a more complex role (1). Monocyte infiltration is quantitatively associated with kidney allograft dysfunction during acute rejection (19). In our analysis, macrophages represent the largest immune cell population in allogeneic grafts, constituting 70.5% of total immune cells.

**Figure 3:**
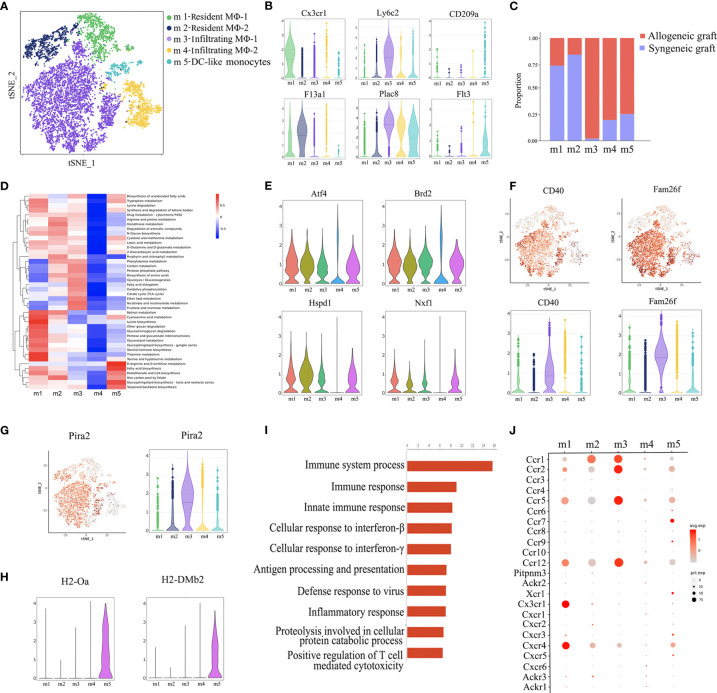
Annotation of macrophage subsets. **(A)** t-SNE plot identifying five distinct populations of macrophages from two allogeneic heart grafts and two syngeneic heart grafts. **(B)** Violin plots displaying the expression of representative markers across macrophage types (Cx3cr1 and F13a1 for resident macrophages, Ly6c2 and Plac8 for infiltrating macrophages, CD209a and Flt3 for dendritic cells). **(C)** Proportion of cells in each macrophage population from allogeneic or syngeneic graft. **(D)** Quantitative Set Analysis for Gene Expression (QuSAGE) analysis examining active cellular metabolic pathways in each macrophage population. **(E)** Violin plots displaying the expression of genes (Atf4, Brd2 Hspd1, Nxf1) associated with cell proliferation, metabolism and activation in each macrophage population. **(F)** t-SNE maps and violin plots indicating the expression of CD40 and Fam26f. **(G)** t-SNE maps and violin plots indicating the expression of Pira2. **(H)** Violin plots displaying the expression of MHC class II molecules H2-Oa and H2-DMb2. **(I)** GO Biological Process and Pathway Analysis of the m3 cluster. **(J)** A dot plot showing the expression of the chemokine receptor in macrophages subsets.

Re-clustering of macrophages revealed five subsets (m1~m5) (**Figure 3A**). Among them, m1 and m2 were characterized as resident macrophages due to the expression of *Cx3cr1* and *F13a1* (20, 21), while m3 and m4 were considered as infiltrating macrophages as indicated by the expression of *Ly6c2* and *Plac8* (22). CD209a is a marker for monocyte-derived dendritic cells (DC) (23) and Flt3 is a key regulator for the development of dendritic cells (**Figure 3B**) (24). Therefore, we annotated m5 as DC-like monocytes. Consistent with previous reports that infiltrating macrophages are associated with rejection, m3 and m4 clusters were predominantly from allogeneic grafts, while m1 and m2 clusters were majorly from syngeneic allografts (**Figure 3C**). Furthermore, on one hand, m1 and m2 adopted M2 macrophage phenotype as shown by a panel of M2 markers, including *Mrc1*, *Folr2* and *Cbr2*, suggesting a potential pro-repair role. On the other hand, m3 and m4 belonged to M1 macrophages as indicated by the expression of M1 proinflammatory chemokines and cytokines, such as *Cxcl10* and *Tnf* (**Supplementary Figure 3**) (25).

Surprisingly, analysis of cellular metabolic pathways demonstrated that different from the other four clusters, the m4 cluster was metabolically “silent” (**Figure 3D**), which was further confirmed by the minimal gene expression of factors related to cell proliferation, metabolism and activation, such as *Atf4*, *Brd2*, *Hspd1* and *Nxf1* (**Figure 3E**) (26), suggesting that m3, not m4, was the subset of infiltrating macrophages actively associated with graft rejection. It is possible that the m4 cluster may be in a transition state, waiting to be activated. In support of this interpretation, the costimulatory receptor CD40 and another membrane molecule Fam26f were expressed at a much higher level in m3 than in m4 (**Figure 3F**). A recent study reported that murine macrophages acquire alloantigen-specific memory, which requires the interaction between MHC-I and PIR-A (27). Interestingly, *Pira2* was highly expressed in m3 (**Figure 3G**). Moreover, the m3 cluster was also characterized by enriched expression of rejection-associated genes, such as *Cxcl9*, *Gzmb*, *Psmb9*, *Isg20*, *Tap1* and *Nampt* (**Supplementary Figure 4**) (28–30). We further performed GO enrichment analysis with the transcriptome of m3 and found enrichment of genes involved in immune response activation, such as immune response, inflammatory response and positive regulation of T cell mediated cytotoxicity (**Figure 3I**). We further analyze the chemokine receptor expression in macrophages, interestingly, Ccr1, Ccr2, Ccr5 and Ccr12 was highly expressed in m3 (**Figure 3J**), and these C-C motif chemokine receptors may contribute to m3 infiltration. Taken together, the m3 cluster was the cell population linked to acute rejection.

Dendritic cells are important antigen presenting cells. From this perspective, we further found that the m5 cluster highly expresses some dendritic cell markers, such as Cd209a and Flt3 (**Figure 3B**), it also highly expressed MHC-II molecules, such as H2-Oa and H2-DMb2 (**Figure 3H**). As shown in **Figure 3C**, the m5 was predominantly composed of cells from allogeneic grafts, it therefore may presents the characteristics of antigen presenting cells.

### Characterization of Endothelial Cells and Identification of a Rejection-Related Cluster

**Figure 4:**
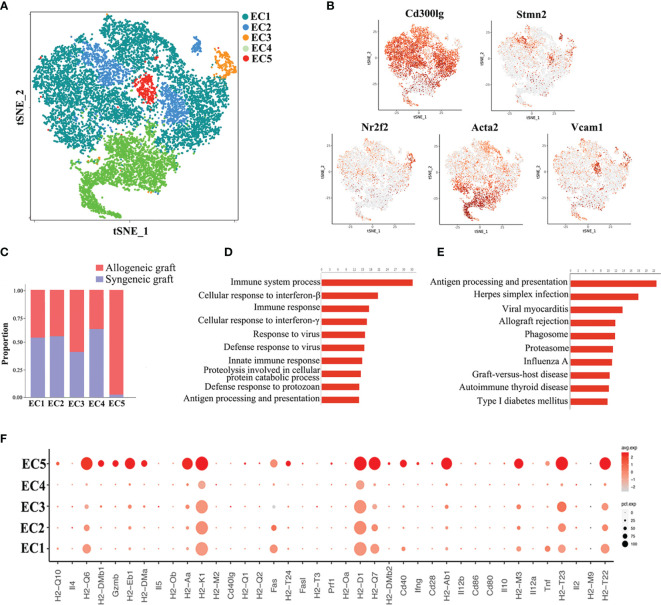
Analysis of endothelial cell (EC) subsets. **(A)** Reclustering of endothelial cells identifying five subsets. **(B)** Expression of canonical cell markers in each EC cluster (EC1-EC5) in the t-SNE plots. **(C)** Proportion of cells in each EC subset from allogeneic or syngeneic graft. **(D)** GO enrichment analysis of EC5. **(E)** KEGG pathway analysis of EC5. **(F)** A dot plot showing the expression of inflammatory genes and MHC class II molecules in each EC subset.

Endothelial cells represented the largest cell population in our analysis, constituting 46.52% of total cells. Re-clustering of all the endothelial cells from both syngeneic and allogeneic grafts unveiled five types (EC1~EC5) (**Figure 4A**). The largest population, EC1, was characterized as capillary endothelial cells due to the expression of *Cd300lg*. EC2 expressed the canonical arterial endothelial cell marker *Stmn2*, while EC3 expressed the venous endothelial cell marker *Nr2f2*. EC4 highly expressed a smooth muscle actin gene *Acta2*, suggesting they were fibroblast-like endothelial cells. The EC5 population expressed *Vcam1*, and likely represented microvascular endothelial cells (**Figure 4B**) (31, 32). Of note, the EC5 cluster was almost exclusively composed of cells from allogeneic grafts (**Figure 4C**). Consistently, GO enrichment analysis and KEGG pathway analysis of EC5 indicated activation of pathways closely related to graft rejection, including antigen processing and presentation, immune system process and allograft rejection (**Figures 4D, E**). More strikingly, a panel of MHC-II molecules (*H2-Aa*, *H2-DMb1*, *H2-Eb1*, *H2-DMa*, *H2-Ab1*, etc.) were predominantly expressed in the EC5 cluster (**Figure 4F**), suggesting that EC5 may exert its function in presenting donor alloantigens to host T cells to mediate transplant rejection.

### Ubd Is Specifically Upregulated in Endothelial Cells During Graft Rejection

**Figure 5:**
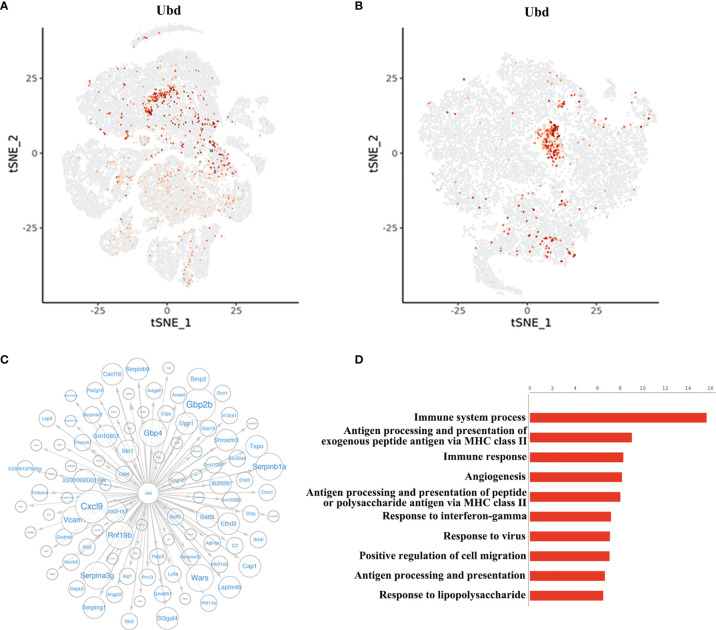
Identification of Ubd in EC5. **(A)** t-SNE map (from **Figure 1A**) indicating the expression of Ubd in all the cells. **(B)** t-SNE plot indicating the expression of Ubd in the re-clustered endothelial cells. **(C)** Correlation network analysis of Ubd displaying its co-expression genes in endothelial cells. **(D)** GO enrichment analysis with Ubd-positive ECs and Ubd-negative ECs.

**Figure 6:**
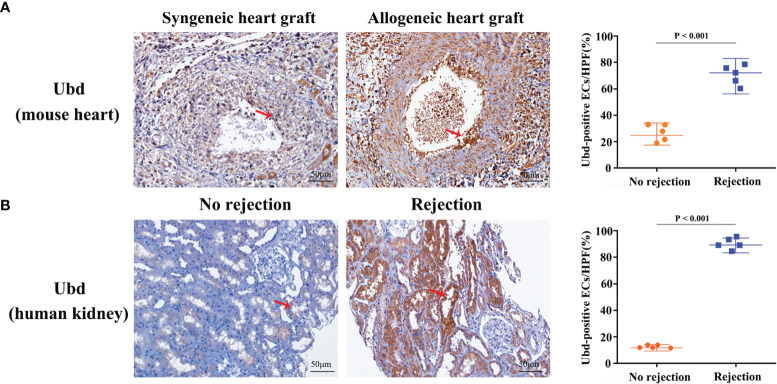
Upregulation of Ubd in endothelial cells during rejection. **(A)** Representative immunohistochemistry staining of Ubd in the allogeneic and syngeneic mouse heart grafts. **(B)** Representative immunohistochemistry staining of Ubd in human kidney biopsy specimens with/without rejection. Arrows indicate positive staining in endothelial cells. Scale bars, 50 mm. Quantitative analysis was shown on the right (n=5 for each group).

Since the cluster EC5 was almost exclusively present in allogeneic graft, we made further analysis of EC5 to identify rejection-specific markers. Excitingly, compared to other cell types or other endothelial cell clusters, only almost all the cells in EC5 highly expressed Ubiquitin D (*Ubd*) gene (**Figures 5A, B**) (33). We next analyzed co-expression genes with *Ubd* and revealed that many of them, such as *Cxcl9*, *Cxcl16*, *Ly6a*, *Batf2*, *Batf3* and *H2-Q2*, were genes which play an important role in regulating immune cells, including T cells, macrophages and DCs (**Figure 5C**). In support of this scenario, pathway analysis indicated the enrichment of genes involved in pathways associated with immune response, such as immune system process, antigen processing and presentation of exogenous peptide antigen *via* MHC class II, and immune response (**Figure 5D**), suggesting Ubd positive endothelial cells activate immune system to mediate graft rejection. The specific upregulation of Ubd in graft rejection was confirmed by immunohistochemistry staining. Ubd was significantly upregulated in endothelial cells in allogeneic heart grafts compared to syngeneic heart grafts (**Figure 6A**).

To investigate clinical relevance, we collected 10 human kidney biopsy specimens (5 with rejection, 5 without rejection, **Supplementary Table 1**). Immunohistochemistry staining showed that massive endothelial cells were Ubd positive in the kidney grafts with rejection. In contrast, staining was sparse or absent in kidney grafts with no injury (**Figure 6B**). These results indicate that Ubd positive cells are closely associated with acute rejection, and Ubd could be considered as a biomarker to predict transplant rejection in the clinic.

## Discussion

This study provides a comprehensive single-cell atlas of gene expression involved in acute rejection using the classical mouse heterotopic heart transplant model. Consistent with previous studies, we confirmed activation and expansion of T cells and NK cells. Moreover, among the five subsets of macrophages we identified, infiltrating macrophages (m3 and m4), rather than resident macrophages (m1 and m2), were associated with graft rejection. Interestingly, metabolic pathway analysis showed that the m4 cluster was in an inactive state. Our results showed m3 and m4 cluster expressed pro-inflammatory chemokines and cytokines Cxcl10 and Tnf, two potent M1 macrophage activation markers. Moreover, the expression of activating molecules, such as *CD40* and *Fam26f*, was elevated in m3, but not in m4. CD40 is a costimulatory molecule, which plays an important role in immune cell activation and survival. Fam26f is a conserved surface molecule, which also modulates immune responses. Specifically, it has been reported that *FAM26F* is an activation marker between resting macrophages and lipopolysaccharide (LPS) activated macrophages, and that the up-regulation of *FAM26F* is related to early liver graft failure (34). Therefore, further studies are required to distinguish the functional and mechanistic difference between m3 and m4, which may shed light on how macrophages are recruited to the allograft and activated. A recent study demonstrated that mouse monocytes and macrophages may acquire alloantigen-specific memory in a PIR-A dependent way. Intriguingly, our analysis also identified the up-regulation of *Pira2* in the m3 cluster, suggesting that the m3 cluster may also function in “trained immunity” as memory cells.

In addition to macrophage surface markers, the m5 cluster also highly expressed MHC-II molecules and presented the characteristics of antigen presenting cells. Previous investigations have shown that monocyte-derived DCs isolated from the allograft function as potent antigen-presenting cells that could drive both T cell proliferation and interferon γ (IFN-γ) production, while depletion of monocyte-derived DCs significantly alleviates rejection (35, 36). Thus, the m5 cluster was also an important group of macrophages involved in rejection. Taken together, the characterization of these macrophage populations provides a valuable framework for studying the role of macrophages in graft rejection and related mechanisms.

Recent studies using single-cell transcriptome profiling have revealed the existence of extensive heterogeneity in ECs in many settings, including tumors, acute lung injury, as well as normal organs, which contribute to the understanding of EC diversity (31, 37, 38). scRNA-seq analysis of a human kidney biopsy specimen identified three endothelial cell groups and one of them was associated with antibody-mediated rejection (8). Our analysis identified 5 populations of ECs (EC1~EC5) in acute rejection. Excitingly, a novel microvascular endothelial cell population (EC5) almost uniquely stemmed from allogeneic heart grafts. In the context of organ transplantation, endothelial cells express both MHC class I and class II molecules, enabling them to present antigens to recipient T cells. Consistent with that, ablation of MHC molecules on ECs mitigates T cell-mediated rejection (39, 40). Interestingly, the allograft-specific EC5 cluster exhibited significant up-regulation of genes involved in processes related to antigen processing and presentation, graft rejection and immune system process. Furthermore, we observed that MHC-II molecules were predominantly expressed in the EC5 cell cluster, suggesting that EC5 is the endothelial cell population that exerts the antigen-presenting capacity. Thus, our investigation broadens the horizon of the role of endothelial cells in transplantation by pinpointing EC5 as the endothelial cell group responsible for rejection.

Since EC5 was uniquely present in allogeneic heart grafts, we performed more analysis to characterize EC5 in order to identify new diagnostic and therapeutic targets. We found that *Ubd* was specifically up-regulated in EC5. Ubd (also called Fat10) is a ubiquitin-like protein that is capable of inducing ubiquitin-independent degradation of proteins *via* the proteasome (33). As for immune responses, Ubd was initially found to be expressed in mature B lymphocytes and dendritic cells, which is involved in antigen presentation and regarded as a biomarker for immune activation (41). In the context of organ transplantation, bioinformatic meta-analysis of microarray datasets and bulk-RNA sequencing indicate that Ubd is related to rejection and has the potential to be considered as a diagnostic marker (42, 43), yet with no confirmation or further study. Our discovery provides a new perspective that Ubd is specifically up-regulated in endothelial cells in acute rejection. Besides, we confirmed the up-regulation of Ubd in endothelial cells in acute rejection in mouse allogeneic hearts and human kidney biopsy specimens. In addition, gene correlation network characterization and functional enrichment analysis suggest that Ubd plays an important role in immune regulation. However, further investigation is needed to address the effect of Ubd expression and why Ubd is specifically induced in EC5, which may shed light on new therapeutic tools to prevent rejection.

In conclusion, we provide a comprehensive landscape of graft-infiltrating cells in acute rejection, as well as their intra-population heterogeneity. We reveal two graft-infiltrating macrophage populations and only one of them is actively involved in graft rejection. Meanwhile, we identify a novel endothelial cell cluster that may potentially exert antigen-presenting capacity to stimulate rejection. Furthermore, the expression of Ubd defines such endothelial cell cluster and could be used as a biomarker for acute rejection.

## Data Availability Statement

The datasets presented in this study can be found in online repositories. The names of the repository/repositories and accession number(s) can be found in the article/**Supplementary Material**.

## Ethics Statement

The studies involving human participants were reviewed and approved by the Research Ethics Committee of the First Affiliated Hospital of Sun Yat-sen University. Written informed consent to participate in this study was provided by the participants’ legal guardian/next of kin. The animal study was reviewed and approved by the Animal Care Ethics Committee of the First Affiliated Hospital of Sun Yat-sen University.

## Author Contributions

YT and XS designed the study and drafted the manuscript. YZ performed mouse heterotopic heart transplantation. YT and JW collected samples. MD carried out immunohistochemistry staining experiments. YT, YZ, JW, XH, CW and XS analyzed and interpreted data. JL, MC, YG and SL provided resources. All authors contributed to the article and approved the submitted version.

## Funding

XS was supported by National Natural Science Foundation of China (31800753) and Guangdong Basic and Applied Basic Research Foundation (2019A1515010639). CW was supported by National Key Research and Development Program of China (2019YFA0111500). YG was supported by National Natural Science Foundation of China (31800758). The project was also funded by Guangdong Provincial Key Laboratory of Organ Donation and Transplant Immunology (2013A061401007, 2017B030314018, 2020B1212060026) and Guangdong Provincial International Cooperation Base of Science and Technology (Organ Transplantation) (2015B050501002, 2020A0505020003).

## Conflict of Interest

The authors declare that the research was conducted in the absence of any commercial or financial relationships that could be construed as a potential conflict of interest.

## Publisher’s Note

All claims expressed in this article are solely those of the authors and do not necessarily represent those of their affiliated organizations, or those of the publisher, the editors and the reviewers. Any product that may be evaluated in this article, or claim that may be made by its manufacturer, is not guaranteed or endorsed by the publisher.
