# Supplementary material for: Single-Cell RNA Sequencing Identifies Intra-Graft Population Heterogeneity in Acute Heart Allograft Rejection in Mouse
Source: Front Immunol. 2022 Feb 10;13:832573. doi: 10.3389/fimmu.2022.832573 (PMC8866760; doi:10.3389/fimmu.2022.832573)
Supplement: Supplementary file 1 [file DataSheet_1.docx]

Supplementary Material


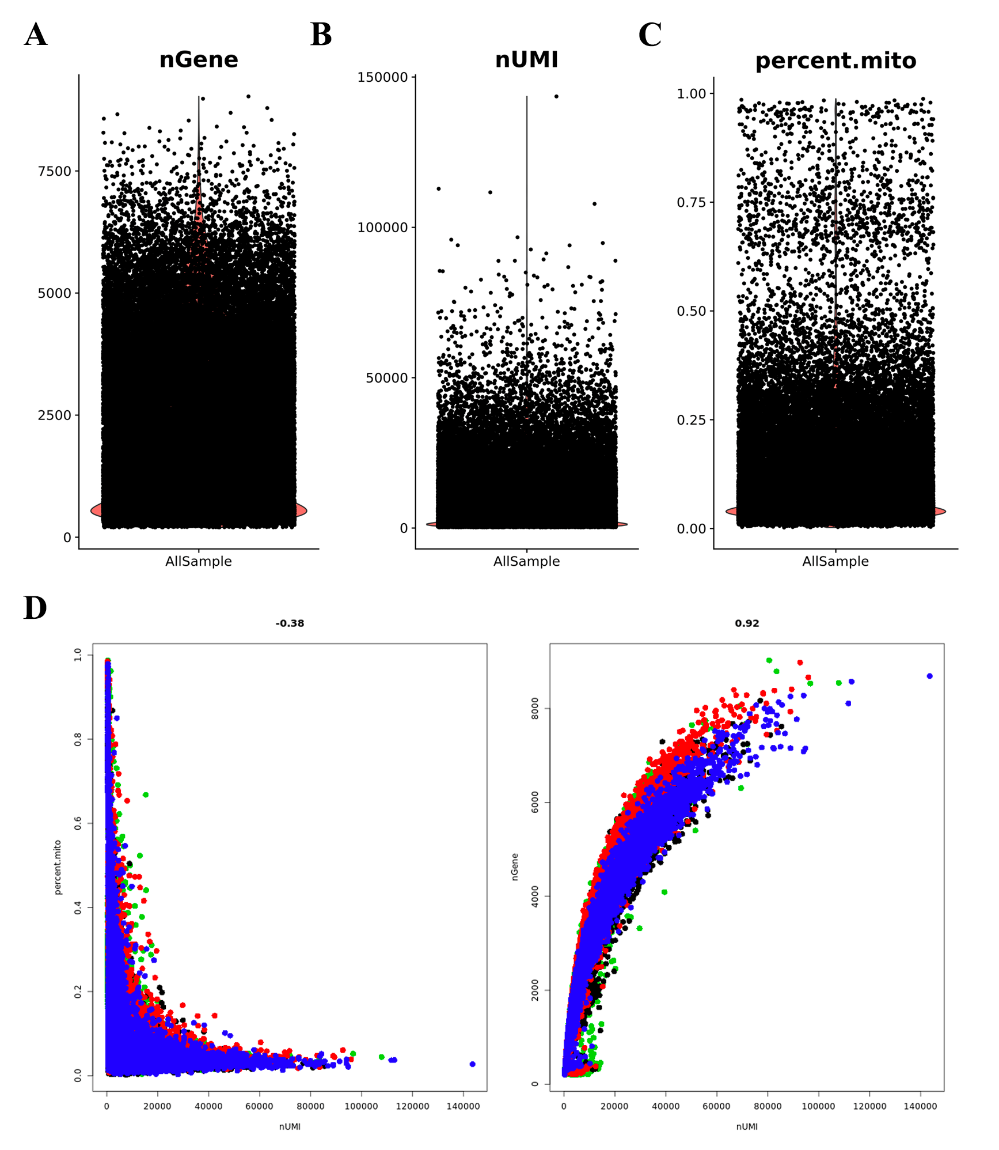


**Supplementary Figure 1.** Quality controls for single-cell RNA-sequencing. (A) Number of genes for single cells. (B) Number of unique molecular identifier (UMI) per cell. (C) Percentage of mitochondrial genes/all genes (expressed as %) within individual single cell transcriptome. (D) Percentage of mitochondrial genes/all genes per cell and total number of genes per cell (nGene) in relation to UML counts are shown.


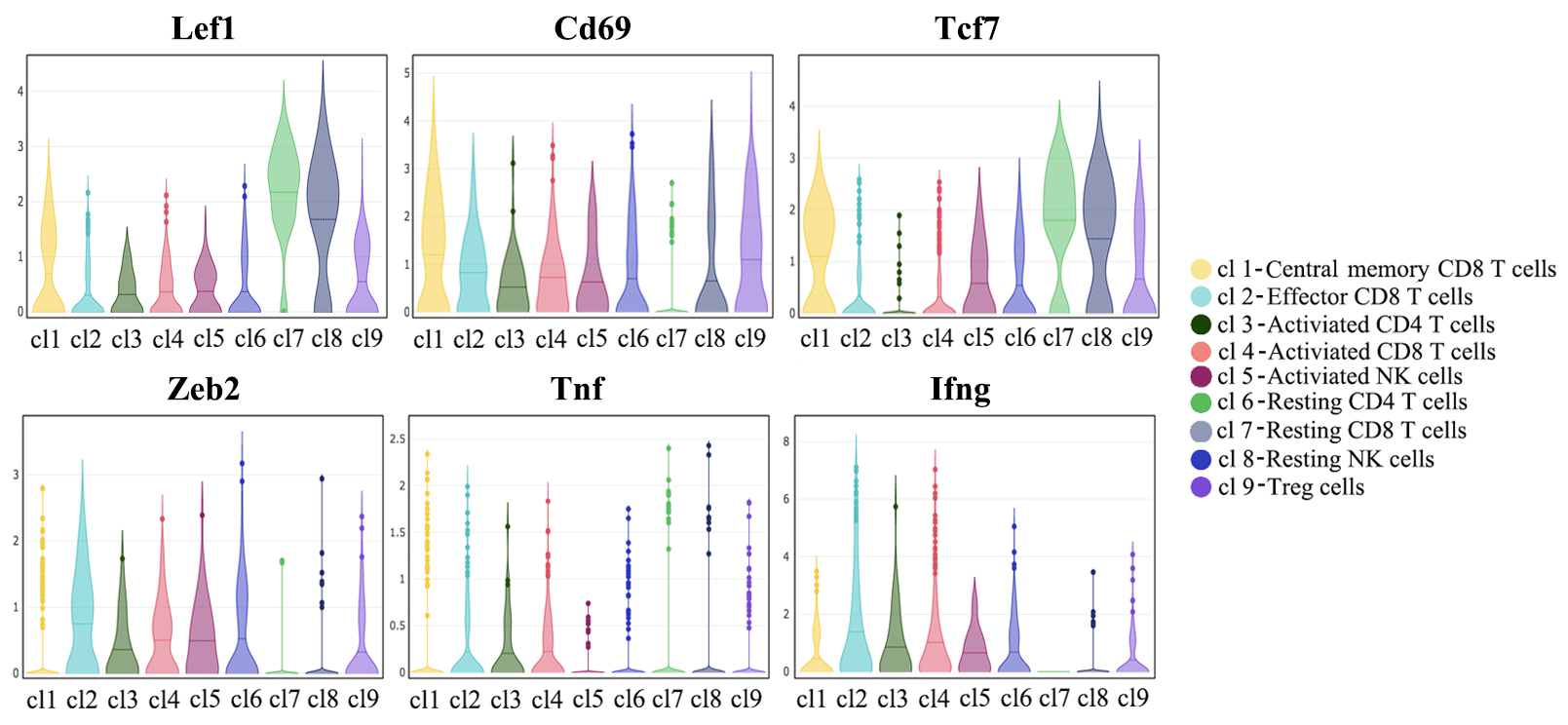


**Supplementary Figure 2.** Violin plots displaying the expression of representative central memory T cells markers (Lef1, Cd69, Tcf7), and effector T cells markers (Zeb2, Tnf, Ifng) in T and NK cell subsets.


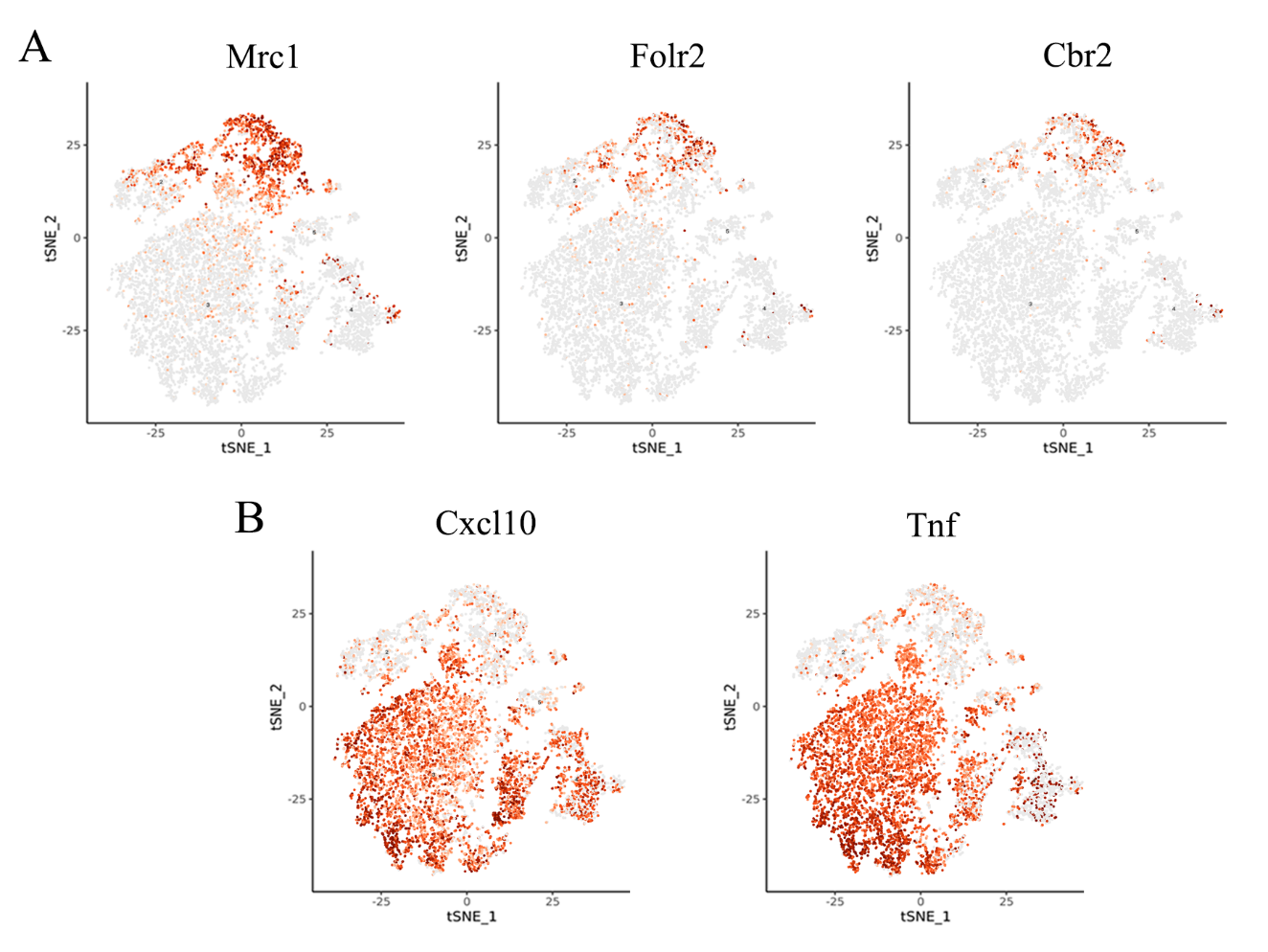


**Supplementary Figure 3.** t-SNE maps indicating the expression of Mrc1, Folr2 and Cbr2, Cxcl10 and Tnf in the five macrophage populations.


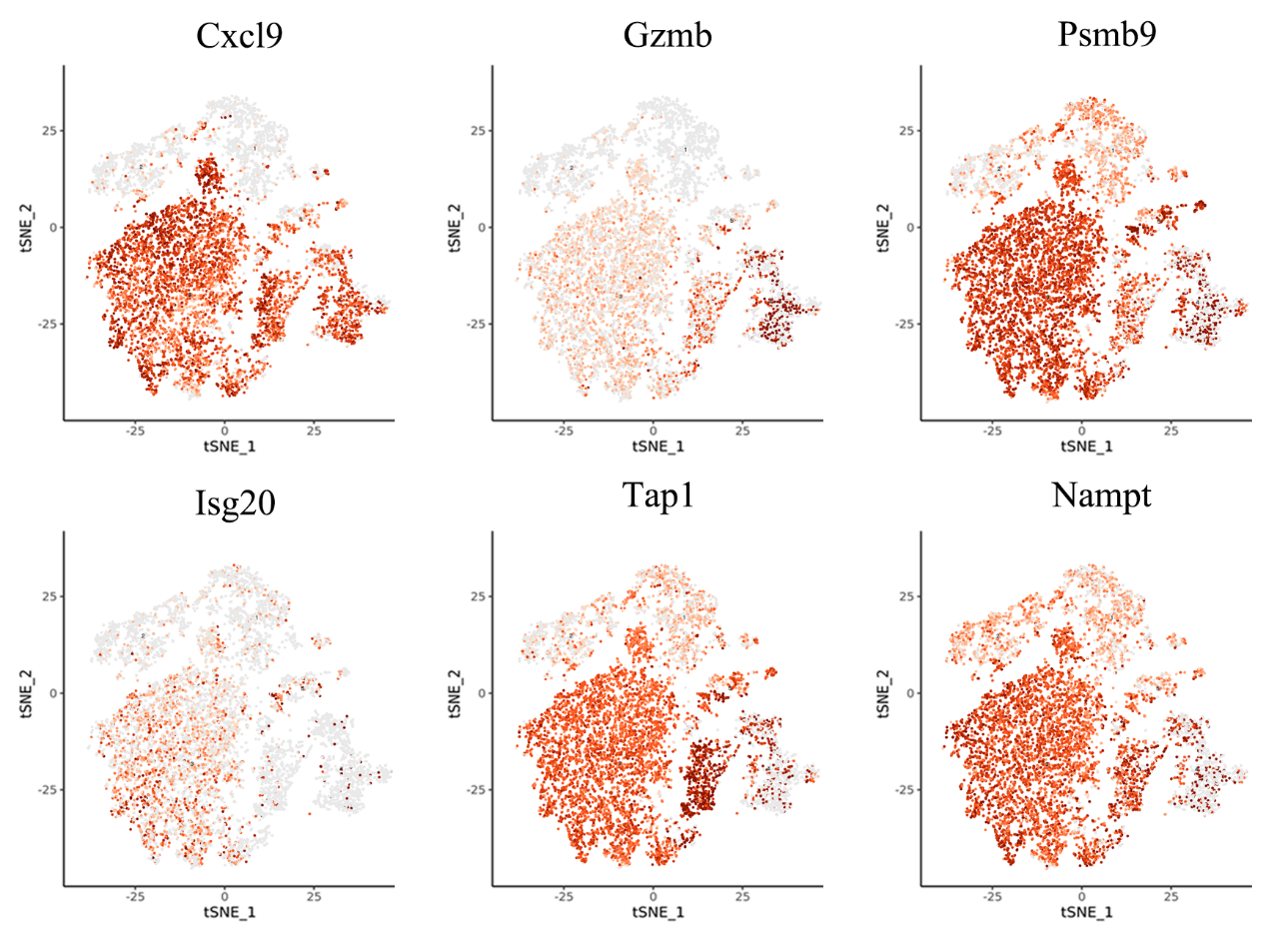


**Supplementary Figure 4.** t-SNE maps indicating the expression of Cxcl9, Gzmb, Psmb9, Isg20, Tap1, Nampt in the five macrophage populations.

| Supplementary Table 1. Baseline characteristics of kidney transplant recipients | | | | | | | | |
| --- | --- | --- | --- | --- | --- | --- | --- | --- |
|  | Donors | | |  | Recipients | | | |
|  | Age  (years) | Gender | Donor type |  | Age  (years) | Gender | Kidney biopsy pathologic diagnosis | Time of rejection  (months) |
| Case 1 | 57 | Female | LD |  | 26 | Male | Rejection | 21 |
| Case 2 | 37 | Male | DBD |  | 56 | Male | Rejection | 2 |
| Case 3 | 22 | Male | DBD |  | 59 | Male | Rejection | 28 |
| Case 4 | 19 | Male | DBD |  | 26 | Male | Rejection | 10 |
| Case 5 | 3 | Male | DCD |  | 11 | Female | Rejection | 34 |
| Case 6 | 56 | Female | LD |  | 29 | Male | No injury | - |
| Case 7 | 46 | Female | LD |  | 25 | Male | No injury | - |
| Case 8 | 50 | Male | LD |  | 26 | Male | No injury | - |
| Case 9 | 48 | Male | LD |  | 46 | Female | No injury | - |
| Case10 | 51 | Female | LD |  | 31 | Male | No injury | - |
| LD: Living Donor; DBD: Donation after brain death; DCD: Donation after circulatory death | | | | | | | | |
